# Supplementary material for: deepTFBS: Improving within‐ and Cross‐Species Prediction of Transcription Factor Binding Using Deep Multi‐Task and Transfer Learning
Source: Adv Sci (Weinh). 2025 May 24;12(30):e03135. doi: 10.1002/advs.202503135 (PMC12376555; doi:10.1002/advs.202503135)
Supplement: Supplementary file 1 — Supporting Information [file ADVS-12-e03135-s001.docx]

**Supplemental Information**

**Supplemental Figure 1**. Standardized computational pipeline for processing DAP-Seq and ChIP-Seq data.

**Supplemental Figure 2**. Impact of class imbalance on deepTFBS-MT performance. (A) Relationship between model performance (PRAUC) and proportion of positive samples in the test dataset for 359 *Arabidopsis* TFs. Each point represents one TF, showing lower PRAUC values generally correspond to TFs with fewer positive samples. (B) The PRAUC of deepTFBS-MT across different TF families.

**Supplemental Figure 3.** Performance comparison of deepTFBS-MT with existing methods with leave-one-chromosome out strategy. Scatter plots showing the comparison of area under ROC curve (AUC) between deepTFBS-MT and (A) deepSEA, (C) DanQ, and (E) BPNet body. Area under PR curve (PRAUC) comparisons between deepTFBS-MT and (B) deepSEA, (D) DanQ, and (F) BPNet body are also shown. Each point represents one TF, with points above the diagonal indicating superior performance by deepTFBS-MT.

**Supplemental Figure 4.** **Genomic distribution and characteristics of predicted regulatory variants.** (A) Genomic distribution of high-impact variants (top 10% by effect score). The stacked bar chart shows the percentage distribution across different genomic features, including promoter regions, UTRs, exons, introns, and intergenic regions. (B) Relationship between variant effect scores and minor allele frequency (MAF). The plot shows decreasing mean MAF with increasing effect scores (binned by log10 scale), suggesting stronger selective constraints on high-impact variants. The dashed line indicates the genome-wide average MAF. (C) Example of a functional variant affecting PR1 gene (AT2G14610) expression. Top: gene structure showing the location of SNP chr2:6321143 (T/A). Bottom: Box plot showing differential gene expression between T (*n*=30) and A (*n*=590) alleles (FDR = 6.92E-4). (D) Differential binding predictions for WRKY transcription factors at the PR1 variant site. Bar plots show binding scores for reference (Ref) and alternate (Alt) alleles across four WRKY family members, demonstrating consistently reduced binding affinity with the alternate allele. (E) The heatmap showing the SNP disrupting the binding motif of the TF *ATSPL9*.

**Supplemental Figure 5**. Relationship between transfer learning improvement and training data size in Arabidopsis and wheat. (A) Correlation analysis between performance improvement and number of binding sites in *Arabidopsis*. The y-axis shows the PRAUC difference between deepTFBS-TL and deepTFBS-ST, while the x-axis shows the log10-transformed number of binding sites. A weak negative correlation (R = -0.13, *P*-value = 0.0024) indicates that transfer learning benefits are slightly more pronounced for TFs with fewer binding sites. (B) Similar correlation analysis for wheat TFs, showing a comparable trend (R = -0.15, *P*-value = 0.1136). The weaker correlation and higher P-value likely reflect the smaller number of TFs available for wheat analysis. In both panels, each point represents one TF, and the blue line indicates the linear regression fit. The negative slopes suggest that transfer learning provides greater benefits when training data is limited, though the relationship is modest.

**Supplemental Figure 6.** Performance comparison of deepTFBS-MT with AgroNT and PDLLM. Scatter plots showing the comparison of area under ROC curve (AUC) between deepTFBS-MT and (A) PDLLM, (C) AgroNT. Area under PR curve (PRAUC) comparisons between deepTFBS-MT and (B) PDLLM and (D) AgroNT. Each point represents one TF, with points above the diagonal indicating superior performance by deepTFBS-MT.

**Supplemental Figure 7.** Quality assessment of WUS DAP-seq data in *Arabidopsis*. (A) Correlation matrix showing the Pearson correlation coefficients between input control and two biological replicates (Ath_rep1 and Ath_rep2). (B) Venn diagram showing the overlap of peaks identified in the two biological replicates. A substantial number of peaks (6,926) were shared between replicates, demonstrating consistency in peak calling. (C-D) Meta-profiles showing the distribution of peak frequencies relative to transcription start sites (TSS) and transcription termination sites (TTS) for replicate 1 (C) and replicate 2 (D). Gray shading indicates 95% confidence intervals. (E) Genomic distribution of WUS binding sites across different functional regions for both replicates. The stacked bar charts show similar distribution patterns between replicates, with predominant binding in promoter and distal intergenic regions.

**Supplemental Figure 8.** Quality assessment of WUS DAP-seq data in maize. (A) Correlation matrix showing the Pearson correlation coefficients between input control and two biological replicates (Zma_rep1 and Zma_rep2). The high correlation between replicates (r = 0.86) indicates good reproducibility. (B) Venn diagram showing the overlap of peaks identified in the two biological replicates. A substantial number of peaks (4,219) were shared between replicates, demonstrating consistency in peak calling. (C-D) Meta-profiles showing the distribution of peak frequencies relative to transcription start sites (TSS) and transcription termination sites (TTS) for replicate 1 (C) and replicate 2 (D). Gray shading indicates 95% confidence intervals. (E) Genomic distribution of WUS binding sites across different functional regions for both replicates. The stacked bar charts show similar distribution patterns between replicates, with predominant binding in promoter and distal intergenic regions.

**Supplemental Figure S9**. Experimental validation of PWM-predicted WUS binding sites using yeast one-hybrid (Y1H) assays.

**Supplemental Figure S10**. Semantic similarity analysis of GO terms enriched in conserved WUS targets.

**Supplemental Figure S11. Overview of the deepTFBS web server interface and functionality.** (A) The database component allows users to query and retrieve predicted binding sites for 512 Arabidopsis and 110 wheat transcription factors. (B) The predictor interface enables users to input DNA sequences for TFBS prediction. Results are presented through an interactive graphical interface highlighting predicted binding sites.

**Supplemental Table S1**. List of 359 *Arabidopsis* transcription factors used for training deepTFBS-MT model.

**Supplemental Table S2**. Performance comparison of different methods (PWM, DeepSEA, DanQ, and deepTFBS-MT) using AUC and PRAUC metrics.

**Supplemental Table S3**. Summary statistics from Fisher’s exact test.

**Supplemental Table** **S4**. Performance evaluation of deepTFBS-TL model across different transcription factors in *Arabidopsis*.

**Supplemental Table** **S5**. Cross-species prediction performance of deepTFBS-TL model for 110 wheat transcription factors.

**Supplemental Table** **S5**. Randomly selected deepTFBS-predicted and PWM-predicted targets using yeast one-hybrid assays.

**Supplemental Table S6**. Training and inference speed of different deep learning frameworks.

**Supplemental Table S7.** Randomly selected deepTFBS-predicted and PWM-predicted targets using yeast one-hybrid assays.

**Supplemental Table** **S8**. Genome-wide WUS binding site predictions and their annotations in *Arabidopsis*.

**Supplemental Table** **S9**. Genome-wide WUS binding site predictions and their annotations in maize.

**Supplemental Table** **S10**. Genome-wide WUS binding site predictions and their annotations in wheat.

**Supplemental Table S11.** Hyperparameters used in deepTFBS framework
